# Supplementary material for: Kondo blockade due to quantum interference in single-molecule junctions
Source: Nat Commun. 2017 May 11;8:15210. doi: 10.1038/ncomms15210 (PMC5437279; doi:10.1038/ncomms15210)
Supplement: Supplementary Information — Supplementary Figures, Supplementary Notes and Supplementary References. [file ncomms15210-s1.pdf]

Supplementary Information

Supplementary Note 1: Single-orbital Anderson model limit

Single molecule junctions that exhibit a zero-bias conductance peak attributed to the Kondo effect are typically modeled using the single-orbital Anderson model,[1]

$$H_{\rm mol} \to H_{\rm AIM} = \sum_{\sigma} \epsilon \ d^{\dagger}_{\sigma} d_{\sigma} + U \ d^{\dagger}_{\uparrow} d_{\uparrow} d^{\dagger}_{\downarrow} d_{\downarrow} \ , \tag{1}$$

and where $H_{\rm hyb} = \sum_{\alpha\sigma}(t_{\alpha} d^{\dagger}_{\sigma} c_{\alpha\sigma} + \text{H.c.})$. This highly simplified model, which entirely neglects the orbital/spatial structure of the molecule, yields a very particular form of the effective Kondo model, Eq. (16), upon Schrieffer-Wolff transformation. For any U and ϵ, one obtains $J_{\rm sd}^2 = J_{\rm ss} J_{\rm dd}$ and $W_{\rm sd}^2 = W_{\rm ss} W_{\rm dd}$, meaning that in the even/odd orbital basis (cf. Eq. (4) of subsection 'Emergent decoupling'), a standard single-channel Kondo model results. The odd channel is strictly decoupled ($J_{\rm o} = 0$) on the level of the bare Hamiltonian, and no multi-channel effects can manifest. This has significant consequences for the physics of a single-orbital model – for example the Kondo temperatures and conductance lineshapes must take a particular form.

The Kondo temperature associated with the single-orbital Anderson model follows from perturbative scaling as,[1]

$$T_{\rm K} \sim (\Gamma U)^{1/2} \exp[\pi\epsilon(\epsilon + U)/\Gamma U] \tag{2}$$

with $\Gamma = \Gamma_{\rm s} + \Gamma_{\rm d}$ and $\Gamma_{\alpha} = \pi\rho_0 t_{\alpha}^2$. In the presence of the gate described by $H_{\rm g}$, one has $\epsilon \to \epsilon - eV_{\rm g}$, yielding the standard quadratic dependence on applied gate, $\ln T_{\rm K}/D \sim (\epsilon - eV_{\rm g})(\epsilon - eV_{\rm g} + U)$. However, note that this behaviour is not necessarily expected in the case of real single-molecule junctions, since nontrivial gate dependences arise in the generic multi-orbital case (as shown explicitly in subsection 'Gate-tunable QI in Kondo-active molecules' for the isoprene junction).

Zero-bias conductance through a single Anderson orbital can be obtained from the Meir-Wingreen formula,[8]

$$G(T) = \frac{2e^2}{h} \ G_0 \int d\omega \ \left(\frac{-\partial f}{\partial \omega}\right) t_{\rm ee}(\omega, T) \ , \tag{3}$$

where f denotes the Fermi function and $t_{\rm ee}(\omega, T) = -\pi\rho_0 \text{Im} \ T_{\rm ee}(\omega, T)$ is the spectrum of the even channel T-matrix. For a single Anderson impurity, $T_{\rm ee}(\omega, T) = (t_{\rm s}^2 + t_{\rm d}^2)\mathcal{G}_{\rm imp}(\omega, T)$,

with $\mathcal{G}_{\rm imp}(\omega,T) = \langle\langle d_\sigma; d_\sigma^\dagger\rangle\rangle$ the full retarded impurity Green's function. The simple form of Supplementary Equation 3 applies in the case of proportionate couplings,[8] automatically fulfilled in the single-orbital case. The source/drain asymmetry factor is given by

$$G_0 = \frac{4\Gamma_{\rm s}\Gamma_{\rm d}}{(\Gamma_{\rm s}+\Gamma_{\rm d})^2} \equiv \frac{4t_{\rm s}^2 t_{\rm d}^2}{(t_{\rm s}^2+t_{\rm d}^2)^2} \equiv \frac{4J_{\rm ss}J_{\rm dd}}{(J_{\rm ss}+J_{\rm dd})^2} \;, \tag{4}$$

which is maximal, $G_0 = 1$, for equal couplings $t_{\rm s} = t_{\rm d}$.

The universal form of $t_{\rm ee}(\omega,T)$ in the Kondo regime gives the universal temperature-dependence of conductance[9] often successfully fit to experimental data.[10,11] Importantly, at particle-hole symmetry $U = -2\epsilon$, the Friedel sum rule[1] pins the T-matrix to $t_{\rm ee}(0,0) = 1$ – meaning that $G(0) = (2e^2h^{-1})G_0$ – characteristic of the Kondo resonance. Note that in this case, the potential scattering terms vanish exactly.[1] This is in fact a consequence of destructive quantum interference between particle and hole processes;[2] conductance is due to the Kondo exchange-cotunneling term alone.

It is instructive to derive the Meir-Wingreen formula Supplementary Equation 3 from the general Kubo formula Eq. (23), since the subsequent generalization to the two-channel case (Sec. S6) provides novel results of relevance to single-molecule junctions. The key step here relies on the fact that the odd conduction electron channel decouples in the single-orbital Anderson model. First, note that the correlator $K(\omega,T) = \frac{1}{2^2}\langle\langle \dot{N}_{\rm s} - \dot{N}_{\rm d}; \dot{N}_{\rm s} - \dot{N}_{\rm d}\rangle\rangle$ can be written as $K(\omega,T) = \frac{1}{(t_{\rm s}^2+t_{\rm d}^2)^2}\langle\langle t_{\rm d}^2\dot{N}_{\rm s} - t_{\rm s}^2\dot{N}_{\rm d}; t_{\rm d}^2\dot{N}_{\rm s} - t_{\rm s}^2\dot{N}_{\rm d}\rangle\rangle$ by current conservation. Using $\dot{N}_\alpha = i[\hat{H},\hat{N}_\alpha]$ with Supplementary Equation 1 we then obtain,

$$K(\omega,T) = -\left(\tfrac{t_{\rm s}t_{\rm d}}{t_{\rm s}^2+t_{\rm d}^2}\right)^2 \sum_{\sigma,\sigma'} \langle\langle f_{{\rm o}\sigma}^\dagger d_\sigma - d_\sigma^\dagger f_{{\rm o}\sigma} \;;\; f_{{\rm o}\sigma'}^\dagger d_{\sigma'} - d_\sigma^\dagger f_{{\rm o}\sigma}\rangle\rangle \;, \tag{5}$$

where $f_{{\rm o}\sigma} = (t_{\rm s}^2+t_{\rm d}^2)^{-1/2}[t_{\rm d}c_{{\rm s}\sigma} - t_{\rm s}c_{{\rm d}\sigma}]$ is the local odd-channel conduction electron operator. Since the odd channel is strictly decoupled from the rest of the system (impurity and even channel), $K(t,T)$ factorizes as,

$$\begin{aligned} K(t,T) = -i\theta(t)\left(\frac{t_{\rm s}t_{\rm d}}{t_{\rm s}^2+t_{\rm d}^2}\right)^2 &\sum_{\sigma,\sigma'} \\ &\Big[\langle d_\sigma(t)d_{\sigma'}^\dagger(0)\rangle_{\rm sys}\times\langle f_{{\rm o}\sigma}^\dagger(t)f_{{\rm o}\sigma'}(0)\rangle_{\rm odd} + \langle d_\sigma^\dagger(t)d_{\sigma'}(0)\rangle_{\rm sys}\times\langle f_{{\rm o}\sigma}(t)f_{{\rm o}\sigma'}^\dagger(0)\rangle_{\rm odd} \\ &-\langle d_\sigma(0)d_{\sigma'}^\dagger(t)\rangle_{\rm sys}\times\langle f_{{\rm o}\sigma}^\dagger(0)f_{{\rm o}\sigma'}(t)\rangle_{\rm odd} + \langle d_\sigma^\dagger(0)d_{\sigma'}(t)\rangle_{\rm sys}\times\langle f_{{\rm o}\sigma}(0)f_{{\rm o}\sigma'}^\dagger(t)\rangle_{\rm odd}\Big] \end{aligned} \tag{6}$$

Some lengthy algebra then yields the following result,

$$\text{Im } K(\omega,T) = \left(\frac{2t_\text{s}^2t_\text{d}^2}{t_\text{s}^2+t_\text{d}^2}\right)\int_{-\infty}^{\infty} d\omega' \text{ Im } \mathcal{G}_\text{imp}(\omega',T)\times[\rho(\omega'-\omega)f(\omega'-\omega)-\rho(\omega'+\omega)f(\omega'+\omega)] \ . \tag{7}$$

In Eq. (23), this gives the ac conductance in terms of the retarded impurity Green function $\mathcal{G}_\text{imp}(\omega,T)$, consistent with the findings of Refs. 7 and 12. Taking the limit $\omega\to 0$ (and assuming flat conduction bands as usual), one recovers the dc conductance in the form of Supplementary Equation 3.

Note, however, that for real molecular junctions involving multiple orbitals where the two-channel description Eq. (16)instead holds, $K(t,T)$ cannot be factorized as in Supplementary Equation 6. In general, both even and odd channels remain coupled to the molecule, and the total conductance then also involves a contribution from the odd channel, requiring to go beyond Supplementary Equation 3.

Supplementary Note 2: Generalized Kondo resonance and derivation of Eq. (5)

We now consider the generalized 2CK model describing off-resonant single-molecule junctions, Eq. (16), focusing on the particle-hole (ph) symmetric case with $\mathbf{W}=\mathbf{0}$. Quantum interference effects give rise to such a potential scattering node, and can in principle be realized in any given system by tuning gate voltages. Generically, the junction is still conducting due to the exchange-cotunneling term, since $J_\text{sd}\neq 0$. The full conductance lineshape $G(T)$, must be obtained numerically from NRG. However, the conductance in the low-temperature limit $G(T\ll T_\text{K})\simeq G(0)$ can be obtained analytically, as shown below.

Combining Eq. (27)and Eq. (28), and noting that $\tilde{\Gamma}_\alpha = 1/(\pi\rho_0)$ and $\mathcal{G}^{(0)}(0)=-i\pi\rho_0$, we have,

$$G(0)=\frac{2e^2}{h}\times|2\pi\rho_0 T_\text{sd}(0,0)|^2 \ . \tag{8}$$

In the even/odd orbital basis (Eq. (4) of subsection 'Emergent decoupling'), $T_\text{sd}(\omega,T) = U_\text{se}U^*_\text{de}T_\text{ee}(\omega,T)+U_\text{so}U^*_\text{do}T_\text{oo}(\omega,T)$, in terms of the T-matrices of the even/odd channels. Importantly, as shown in subsection 'Emergent decoupling', the odd channel decouples asymptotically. The molecule undergoes a Kondo effect with the even channel since $J_\text{e} > J_\text{o}$; this

cuts off the RG flow with the odd channel and effectively disconnects it. This is an emergent low-energy phenomenon, not a property of the bare system. In all cases, therefore, we have $T_{\rm oo}(0,0)=0$, meaning that,

$$G(0)=\frac{2e^2}{h}\times|2\pi\rho_0\;U_{\rm se}U^*_{\rm de}T_{\rm ee}(0,0)|^2\;. \tag{9}$$

Furthermore, at ph symmetry, $i\pi\rho_0T_{\rm ee}(0,0)=t_{\rm ee}(0,0)=1$ due to the Kondo effect, and so

$$G(0)=\frac{2e^2}{h}\times|2U_{\rm se}U^*_{\rm de}|^2=\frac{2e^2}{h}\times\frac{4J^2_{\rm sd}}{4J^2_{\rm sd}+(J_{\rm ss}-J_{\rm dd})^2}\;. \tag{10}$$

Supplementary Equation 10 generalizes the Meir-Wingreen result for the single-orbital Anderson model, Supplementary Equation 4, to the generic multi-orbital (molecular junction) case, and reduces to it when $J^2_{\rm sd}=J_{\rm ss}J_{\rm dd}$.

Supplementary Note 3: Non-equilibrium conductance and derivation of Eq. (7)

In the special case $J_{\rm e}=J_{\rm o}$, the effective 2CK model Eq. (16), is precisely at a frustrated quantum critical point.[13,14] In practice, $J_{\rm e}=J_{\rm o}$ requires both $J_{\rm sd}=0$ and $J_{\rm ss}=J_{\rm dd}$, and is therefore not expected to be relevant to real molecular junction systems. However, $\delta=\frac{1}{2}(J_{\rm e}-J_{\rm o})$ could still be small, especially near a quantum interference node in $J_{\rm sd}$. When $\delta^2=J^2_{\rm sd}+J^2_{-}<T_{\rm K}$, 2CK quantum critical fluctuations control the junction conductance. Interestingly, exact analytic results can be obtained in this special regime, including the non-linear conductance away from thermal equilibrium. Similar calculations have been performed for the two-impurity Kondo model in Supplementary Reference 15 and for charge-Kondo quantum dot devices in Supplementary Reference 16.

The source of the exact results is the Emery-Kivelson solution[17] of the regular 2CK model at the Toulouse point.[18] By using bosonization methods, Supplementary Reference 17 showed that a spin-anisotropic generalization of the 2CK model drastically simplifies to a Majorana resonant level at a special point in parameter space – the Toulouse point (analogous to a similar spin-anisotropic point for the single-channel Kondo problem[18]). In Supplementary Reference 19 Schiller and Hershfield then obtained the nonequilibrium serial conductance at this Toulouse point, exploiting the fact that the effective Majorana resonant level model is non-interacting and exactly solvable.

Of course, physical systems are not near the Toulouse point, and therefore conductance lineshapes are in general different from those obtained using the Emery-Kivelson solution. Importantly, however, the 2CK critical point has an emergent spin isotropy.[14] This means that properties of the critical point are independent of any spin-anisotropy in the bare model. Exploiting the RG principle that the flow from high to low energies has no memory, one can argue that subsequent low-energy crossovers (due to perturbations to the critical point $\delta \neq 0$), are also independent of spin-anisotropy in the bare model. In particular, the same low-energy crossover must occur in the physical spin-isotropic model as at the Toulouse point. Therefore the Toulouse limit solution can be used for the low-temperature crossover – provided there is good scale separation $T^* \ll T_{\mathrm{K}}$ (where $T^* \sim \delta^2$ is the Fermi liquid crossover scale[14] generated by relevant perturbations J_{sd} and/or J_-).

Following Supplementary Reference 19, we obtain Eq. (7) of subsection 'Kondo resonance'. The precise quantitative agreement between the predicted $G(T)$ at linear response and NRG results (see Figure 3b) validate the above lines of argumentation.

Supplementary Note 4: Kondo blockade and derivation of Eq. (8)

We now consider the case of a quantum interference node $J_{\mathrm{sd}} = 0$. Conductance through the molecular junction is mediated only by W_{sd} (for simplicity, we again take the ph-symmetric case $W_{\mathrm{ss}} = W_{\mathrm{dd}} = 0$). In general, $J_{\mathrm{ss}} \neq J_{\mathrm{dd}}$, meaning that a Kondo effect will develop with the more strongly coupled lead. For concreteness, we take now $J_{\mathrm{ss}} > J_{\mathrm{dd}}$. The drain lead therefore decouples for $T \ll T_{\mathrm{K}}$. As shown below, this produces an exact node in the total conductance $G(T = 0) = 0$. The perturbative result for the conductance $G(T \gg T_{\mathrm{K}}) \sim W_{\mathrm{sd}}^2$, valid at high temperatures, is quenched at low temperatures due to interactions and the Kondo effect.

First, note that the local retarded electron Green's function of the more strongly coupled lead vanishes at $T = \omega = 0$ due to the Kondo effect, $\mathcal{G}_{\mathrm{ss}}(0,0) = 0$. This follows from the T-matrix equation, Eq. (28), using $\mathcal{G}^{(0)}(0) = -i\pi\rho_0$ and $i\pi\rho_0 T_{\mathrm{ss}}(0,0) = t_{\mathrm{ss}}(0,0) = 1$,

$$\mathcal{G}_{\mathrm{ss}}(0,0) = -i\pi\rho_0[1 - i\pi\rho_0 T_{\mathrm{ss}}(0,0)] = 0 \ . \tag{11}$$

This depletion of the lead electron density at the molecule is due to the Kondo effect, and arises only for $T \ll T_{\mathrm{K}}$. The conductance through the molecule is therefore blocked because

there are no available source lead states to facilitate transport. Formally, this is proved from the optical theorem, assuming that the low-energy physics can be understood in terms of a renormalized non-interacting system: the conductance (at $T = 0$) is then related to the total reflectance $G(0) = (2e^2h^{-1})[1 - |r|^2]$, where[6] $r = 1 - 2\tilde{\Gamma}_{\rm s}\mathcal{G}_{\rm ss}(0,0)$.

Alternatively, we can use the Kubo formula, Eq. (23)-Eq. (5)to obtain an expression for the conductance without resorting to Fermi liquid theory. In this regime with $J_{\rm sd} = 0$ but $W_{\rm sd} \neq 0$, we have $i\hat{\Omega} = W_{\rm sd}\sum_\sigma(c^\dagger_{{\rm s}\sigma}c_{{\rm d}\sigma} - {\rm H.c.})$. Since at $T = 0$ the drain channel is decoupled, $K(t,T)$ factorizes as in Supplementary Equation 6. Following the same steps as in Sec. S4, we finally obtain $G(0)$ in terms of the retarded electron Green's function $\mathcal{G}_{\rm ss}(0,0)$,

$$G(0) = \frac{2e^2}{h} \times 4i\pi\rho_0 W_{\rm sd}^2\mathcal{G}_{\rm ss}(0,0) = \frac{2e^2}{h} \times (2\pi\rho_0 W_{\rm sd})^2[1 - t_{\rm ss}(0,0)] = 0\ . \qquad (12)$$

The total conductance at $T = 0$ therefore exactly vanishes since $t_{\rm ss}(0,0) = 1$. Note also that the high-temperature perturbative result is precisely recovered if one sets $t_{\rm ss} = 0$.

One might wonder whether the suppression of conductance due to quantum interference in Kondo-active molecular junctions is related to the Fano effect[20–22] observed e.g. in STM experiments of single magnetic impurities on metallic surfaces. In fact the mechanisms are very different – the Kondo blockade arising here is a novel phenomenon. The Fano effect arises simply because electronic tunneling in an STM experiment can take two pathways – either into a magnetic impurity, or directly into the host metal. The quantum interference is completely on the level of the effective hybridization and is a non-interacting effect. There is no intrinsic quantum interference on the interacting impurity. In the Fano effect, the effective Kondo model must always have $J_{\rm sd} > 0$, and the Kondo effect therefore involves conduction electrons in both the host metal and the STM tip. Furthermore, the problem can always be cast in terms of a single effective channel with asymmetric density of states, yielding asymmetric lineshapes. Note that one also obtains Fano-like lineshapes for trivial resonant level defects with no interactions.

By contrast, there is no direct source-drain conductance pathway in single-molecule junction devices – cotunneling proceeds only throughs the molecule, and $J_{\rm sd} = 0$ arises due to intrinsic quantum interference (i.e., a characteristic property of the isolated molecule and its contacting geometry). The Kondo blockade is an exact conductance node of the strongly

interacting system at $T = 0$, which arises because the molecule is asymptotically bound to only one of the two leads – its Kondo cloud is impenetrable at low energies to cotunneling embodied by W_{sd}. At higher temperatures, the conductance $G(T) \sim W_{\mathrm{sd}}^2$ is 'blind' to the quantum interference node in J_{sd}; the Kondo blockade arises entirely from the interplay between intrinsic molecular quantum interference and Kondo physics. The Kondo blockade lineshape is particle-hole symmetric and a universal function of T/T_{K}.

In the context of double quantum dots realizing a side-coupled two-impurity Kondo model, conductance can also be suppressed.[22] However, this arises because two spin-$\frac{1}{2}$ quantum impurities are successively screened by a single conduction electron channel in a two-stage process. This mechanism is not related to the Kondo blockade, which involves a net spin-$\frac{1}{2}$ molecule and single-stage Kondo screening by two conduction electron channels.

Supplementary Note 5: Cotunneling amplitudes

The benzyl radical in Figure 5(a) of subsection 'Gate-tunable QI in Kondo-active molecules' comprises 7 carbons in a planar arrangement, all sp^2 hybridized [formally (λ^3-methyl)-$2\lambda^2, 5\lambda^3$-benzene], while the isoprene-like molecule in Figure 5(d) involves 5 sp^2 hybridized carbons [formally 2-(λ^3-methyl)-$4\lambda^3$-buta-1,3-diene]. The extended π system of each is described by the Pariser-Parr-Pople model, using the standard Ohno parametrization.[4] As input to the NRG calculations, we computed the effective 2CK model parameters from Eq. (19)and Eq. (20)as a function of gate voltage eV_{g}. These are shown in Supplementary Figures 1 and 2.

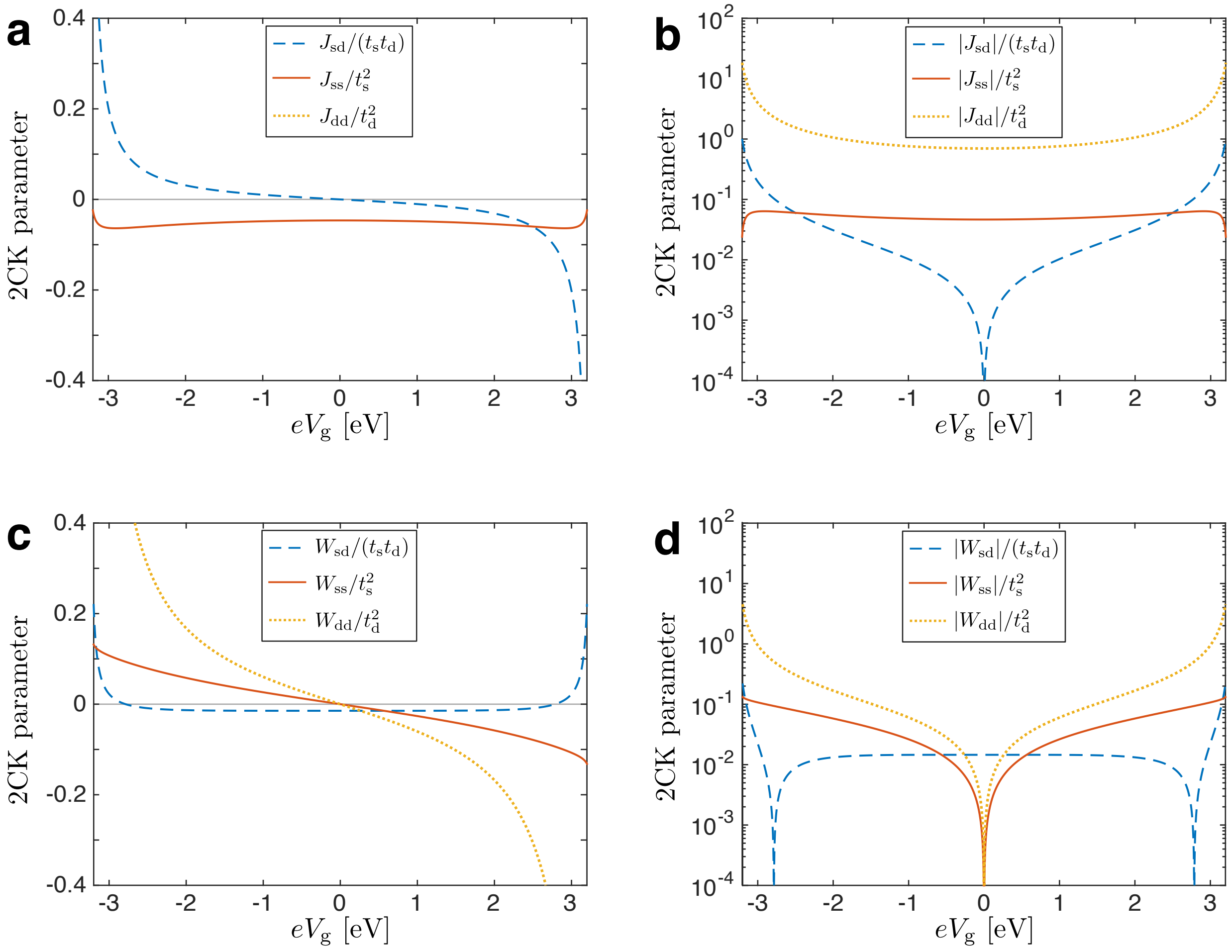

Supplementary Figure 1. **Effective 2CK parameters for the benzyl molecule.**
(**a,b**) Dimensionless exchange coupling $J_{\alpha\alpha'}/(t_\alpha t_{\alpha'})$; (**c,d**) dimensionless potential scattering $W_{\alpha\alpha'}/(t_\alpha t_{\alpha'})$. A linear(logarithmic) scale is used for panels **a** & **c** (**b** & **d**). Computed for $t_s = t_d$. Note that J_{sd} has a single node at $eV_g = 0$.

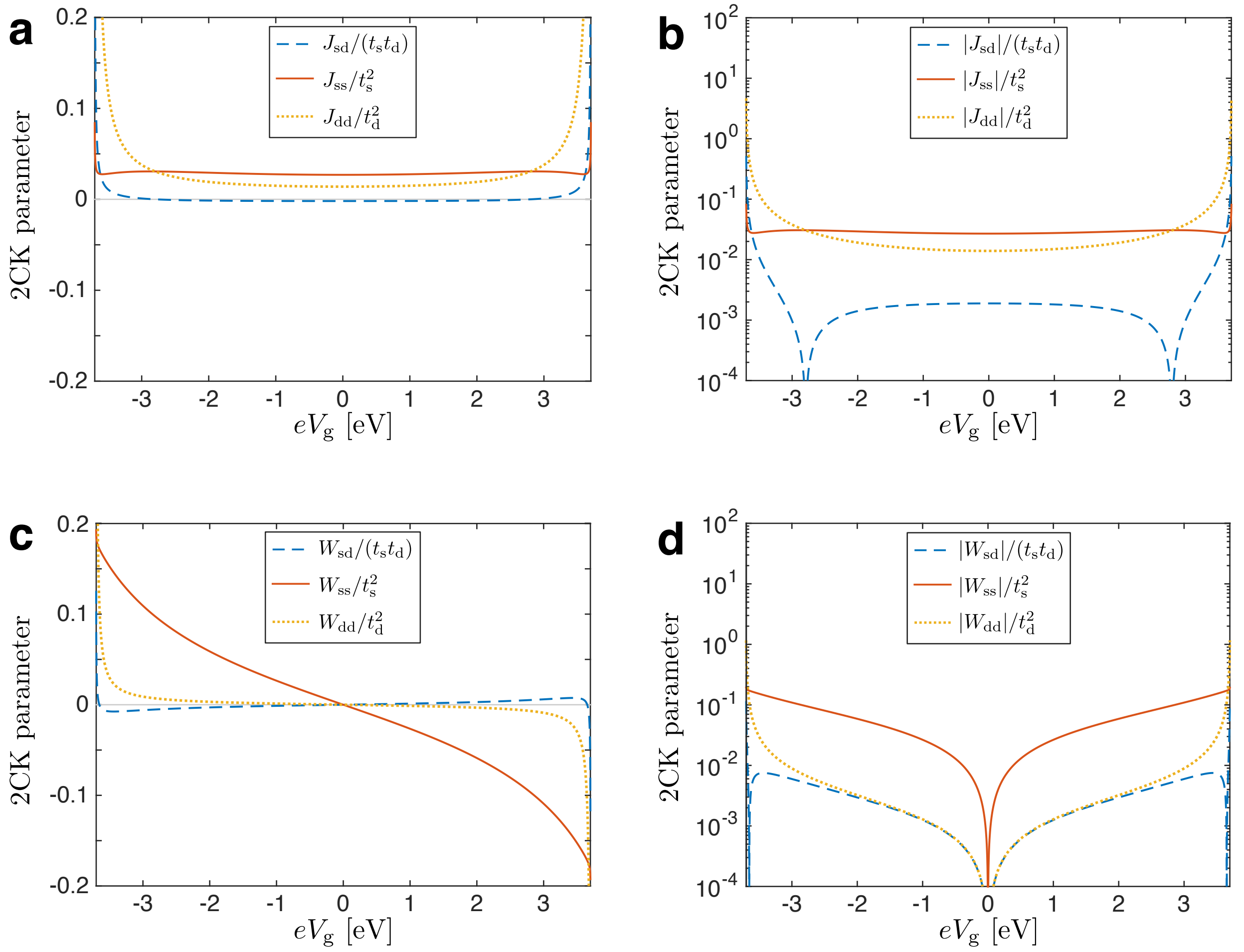

Supplementary Figure 2. **Effective 2CK parameters for the isoprene-like molecule.** (**a,b**) Dimensionless exchange coupling $J_{\alpha\alpha'}/(t_\alpha t_{\alpha'})$; (**c,d**) dimensionless potential scattering $W_{\alpha\alpha'}/(t_\alpha t_{\alpha'})$. A linear(logarithmic) scale is used for panels **a** & **c** (**b** & **d**). Computed for $t_s = 6.17 t_d$. Note that J_{sd} has two nodes at finite gate voltage.

Supplementary References

[1] AC Hewson, "The Kondo problem to heavy fermions," Cambridge University Press (1997).

[2] KGL Pedersen, M Strange, M Leijnse, P Hedegård, GC Solomon, and J Paaske, "Quantum interference in off-resonant transport through single molecules," Physical Review B **90**, 125413 (2014).

[3] R Bulla, TA Costi, and T Pruschke, "Numerical renormalization group method for quantum impurity systems," Reviews of Modern Physics **80**, 395 (2008).

[4] K Ohno, "Some remarks on the Pariser-Parr-Pople method," Theoretica Chimica Acta **2**, 219–227 (1964).

[5] A Weichselbaum and J von Delft, "Sum-rule conserving spectral functions from the numerical renormalization group," Physical Review Letters **99**, 076402 (2007).

[6] A Oguri and AC Hewson, "NRG approach to the transport through a finite Hubbard chain connected to reservoirs," Journal of the Physical Society of Japan **74**, 988–996 (2005).

[7] AI Tóth, L Borda, J von Delft, and G Zaránd, "Dynamical conductance in the two-channel Kondo regime of a double dot system," Physical Review B **76**, 155318 (2007).

[8] Y Meir and NS Wingreen, "Landauer formula for the current through an interacting electron region," Physical Review Letters **68**, 2512 (1992).

[9] TA Costi, AC Hewson, and V Zlatic, "Transport coefficients of the Anderson model via the numerical renormalization group," Journal of Physics: Condensed Matter **6**, 2519 (1994).

[10] D Goldhaber-Gordon, J Göres, MA Kastner, H Shtrikman, D Mahalu, and U Meirav, "From the Kondo regime to the mixed-valence regime in a single-electron transistor," Physical Review Letters **81**, 5225 (1998).

[11] GD Scott and D Natelson, "Kondo resonances in molecular devices," ACS Nano **4**, 3560–3579 (2010).

[12] M Sindel, W Hofstetter, J Von Delft, and M Kindermann, "Frequency-dependent transport through a quantum dot in the Kondo regime," Physical Review Letters **94**, 196602 (2005).

[13] Ph Nozieres and A Blandin, "Kondo effect in real metals," Journal de Physique **41**, 193–211 (1980).

[14] I Affleck and AWW Ludwig, "Exact conformal-field-theory results on the multichannel Kondo effect: Single-fermion greens function, self-energy, and resistivity," Physical Review B **48**, 7297

(1993).

[15] E Sela and I Affleck, “Nonequilibrium transport through double quantum dots: Exact results near a quantum critical point,” Physical Review Letters **102**, 047201 (2009).

[16] AK Mitchell, LA Landau, L Fritz, and E Sela, “Universality and scaling in a charge two-channel Kondo device,” Physical Review Letters **116**, 157202 (2016).

[17] VJ Emery and S Kivelson, “Mapping of the two-channel Kondo problem to a resonant-level model,” Physical Review B **46**, 10812 (1992).

[18] G Toulouse, “Infinite-U Anderson Hamiltonian for dilute alloys,” Physical Review B **2**, 270 (1970).

[19] A Schiller and S Hershfield, “Exactly solvable nonequilibrium Kondo problem,” Physical Review B **51**, 12896 (1995).

[20] O Újsághy, J Kroha, L Szunyogh, and A Zawadowski, “Theory of the fano resonance in the STM tunneling density of states due to a single Kondo impurity,” Physical Review Letters **85**, 2557 (2000).

[21] A Schiller and S Hershfield, “Theory of scanning tunneling spectroscopy of a magnetic adatom on a metallic surface,” Physical Review B **61**, 9036 (2000).

[22] R Žitko, “Fano-Kondo effect in side-coupled double quantum dots at finite temperatures and the importance of two-stage Kondo screening,” Physical Review B **81**, 115316 (2010).
